# Effects of OsteoStrong vs. dynamic multicomponent exercise on physical function in older women in the BONEMORE randomized controlled trial

**DOI:** 10.1007/s40520-026-03421-4

**Published:** 2026-07-05

**Authors:** Peter W.S. Lindberg, Hans Ranch Lundin, Eva Andersson, Ann-Charlotte Grahn Kronhed, Eva Toth-Pal, Maria Sääf, Christina Kaijser Alin, Helena Salminen

**Affiliations:** 1 Department of Neurobiology, Care Sciences and Society, Karolinska Institutet, Stockholm, 171 77 Sweden; 2 Department of Physical Activity and Health, Swedish School of Sport and Health Sciences, Stockholm, Sweden; 3 Department of Molecular Medicine and Surgery, Karolinska Institutet, Stockholm, Sweden; 4 Department of Health, Medicine and Caring Science, Linköping University, Linköping, Sweden; 5 Academic Healthcare Centre Stockholm, Stockholm, Sweden

**Keywords:** Osteoporosis, Osteopenia, Dynamic exercise, Isometric exercise, Postmenopausal women, Physical function, Muscle strength, Balance

## Abstract

**Background:**

Limited research exists on the effects of OsteoStrong on physical function in older women.

**Aim:**

This randomized controlled trial aimed to evaluate and compare the effects of OsteoStrong (OS) and dynamic multicomponent exercise (DME) on functional outcomes in older women with osteopenia or osteoporosis.

**Methods:**

A total of 194 women aged 65–79 years with a T-score of ≤–1.0 at the hip and/or spine were randomized to nine months of either OS (once weekly, 20 min) or DME (twice weekly, 60 min). Outcomes included measures of muscle strength (hand grip and back strength), back and trunk endurance, mobility (sit-to-stand tests, gait speed, Timed Up and Go), and balance (one-leg standing time, tandem standing, tandem walking). Measurements were conducted at baseline and again at nine months.

**Results:**

Both OS and DME showed significant improvements in grip strength, back strength, isometric trunk flexion endurance, gait speed 30 m (m/sec), 5 sit-to-stand (sec) and 50 sit-to-stand speed (n/sec). DME resulted in greater improvements in gait speed 30 m (m/sec) (+ 7.1% vs. +3.2%, p = 0.001), isometric trunk extension (+ 27.6% vs. +4.4%, p = 0.007), and one-leg standing balance (right leg: +13.1% vs. -2.1%, p = 0.001; left leg:+13.3% vs. -2.4%, p = 0.001) compared to OS, while no significant between-group differences were observed for the remaining outcomes.

**Conclusion:**

These findings suggest that while both OS and DME improve physical function in older women with osteopenia or osteoporosis, DME provides superior benefits in gait speed, back muscle endurance, and balance. These findings should be interpreted with caution, as they are based on secondary outcomes.

**Supplementary Information:**

The online version contains supplementary material available at 10.1007/s40520-026-03421-4.

## Introduction

International guidelines emphasize exercise as a fundamental component in osteoporosis management [1, 2]. One of the most important benefits of exercise is its ability to improve muscle function and balance. In particular, dynamic multicomponent exercise (DME), which combines various types of exercise within the same session, has been shown to maintain or increase bone mineral density (BMD), enhance physical function, reduce fall risk, and improve health-related quality of life [3–6]. While dynamic exercise (i.e. muscle contraction with visible joint movement) has been extensively studied, research on isometric exercise (i.e. muscle contraction without any visible joint movement) remains limited. Although DME results in positive health benefits, the physical demands and complexity of DME may pose challenges for older adults, particularly those with mobility or balance impairments, highlighting the need for alternative types of exercise. Isometric exercise may offer a potential alternative for individuals who have difficulties performing dynamic exercise. Previous research has demonstrated that isometric exercise may improve muscle strength [7, 8]. However, there is a need for more research specifically aimed at older adults with osteopenia or osteoporosis on whether the effects of isometric exercise are comparable to dynamic exercise. Dynamic and isometric exercises influence musculoskeletal health through mechanotransduction, where muscle activation stimulates cellular responses in bone and muscle tissue [9]. While dynamic exercises generate cyclical loading, isometric contractions may induce sufficient mechanical strain to promote bone formation and enhance neuromuscular function [8, 10].

A relatively new type of isometric exercise is machine-based isometric axial loading, where the user performs isometric exercises with the use of special machines. OsteoStrong^®^, a company from the United States of America, has developed a machine-based training system consisting of four machines that facilitate low-impact, high-intensity isometric axial loading. The training system is supposed to improve various health outcomes, including bone and muscle strength, balance, posture, and pain reduction [11]. However, research investigating the effectiveness of OsteoStrong^®^ is limited. Further investigation is needed to determine whether OsteoStrong^®^ can serve as an alternative or complement to recommended multicomponent dynamic exercise in osteoporosis management. The aim of this study was to compare the effect of OsteoStrong^®^ (OS) and dynamic multicomponent exercise (DME) on muscle function, mobility and balance performance in older women with osteopenia or osteoporosis. The findings of this study will increase our knowledge of the effectiveness of OS compared to DME on physical functions.

## Materials and methods

### Study design

This two-arm randomized controlled trial (RCT) was conducted in Stockholm, Sweden. Eligible participants were assigned to either nine months of OS or DME. This study presents secondary analyses based on data from an RCT primarily designed to assess bone strength as the main outcome. The study protocol is registered at ClinicalTrials.gov (ID: NCT05721014).

### Participants

Postmenopausal women aged 65 to 79 years were recruited between September 2021 and June 2022 through newspaper advertisements, a women’s organization, and Facebook. Potential participants underwent eligibility screening. The inclusion criteria included a T-score ≤–1.0 at the hip and/or spine, vaccination against COVID-19, and the ability to participate in the training program for nine months. Exclusion criteria were as follows: ongoing bone-specific drug treatments initiated within the last year, or previous treatments discontinued within the last five years; delays in ongoing denosumab treatment exceeding eight months or bisphosphonates exceeding 18 months; ongoing treatment with oral corticosteroids (≥ 5 mg Prednisolone); vertebral fractures diagnosed within the last three months that were not assessed by a physician or treated with bone-specific drugs; bilateral hip replacement; symptomatic disc herniation, inguinal or umbilical herniation; untreated hypertension; other conditions that could impact study results or participation (e.g., malignant diseases, secondary osteoporosis, or muscular dystrophy); conditions preventing the impact microindentation test (e.g., large edema, skin infection, or allergy to local anesthetics); or ongoing or previous training at OsteoStrong™. Participants began the exercise program at six different time points, depending on their recruitment date.

### Randomization and blinding

Eligible participants were randomized using a stratified randomization process that accounted for the presence or absence of current bone-specific drug therapy. A computer-generated block randomization sequence, with block sizes varying between 4 and 8, was prepared and placed into envelopes by a researcher who was not involved in participant assessments. Participants were allocated to groups by researchers after baseline data collection. The researchers responsible for assigning participants were blinded to group allocations during the baseline assessments but not during follow-up. All baseline measurements were conducted by two blinded physical therapists. However, it was not feasible to blind participants or the training instructors delivering the interventions.

### Interventions

#### Treatment arm A: OsteoStrong^®^ (OS)

The OS sessions were conducted once a week at OsteoStrong Solna (Stockholm County) following the OS concept, which involves high-intensity isometric axial bone loading using four machines (Spectrum™): the “upper growth trigger” (chest press), “lower growth trigger” (leg press), “core growth trigger” (pull-down), and “postural growth trigger” (deadlift). Each session also included balance exercises on vibration platforms (Power Plate™), such as one-leg standing. All participants received a 60-minute introductory session based on the OsteoStrong™ concept. During the OS, participants applied isometric force by pressing or pulling on the machines for approximately 15–20 s per machine. The target forces (called “trigger levels”) were 2.5 times their body weight on the upper and postural growth triggers, 1.5 times on the core growth trigger, and 4.2 times on the lower growth trigger [12, 13]. The instructor monitored the exercises using the visible display on each machine, which provided real-time feedback by showing the participants’ generated force on a diagram. This allowed participants to receive immediate feedback on their performance. The total time allocated for the exercise program was 20 min, although sessions could be completed in less time. The exercises were progressively adjusted over time to match the participants’ capabilities. After each session, participants had the option to use a massage chair.

#### Treatment arm B: dynamic multicomponent exercise (DME)

The DME program was designed based on current exercise recommendations for individuals with osteoporosis and included strength training, balance training, and weight-bearing exercises [14]. The DME was conducted twice a week at The Swedish School of Sport and Health Sciences in Stockholm. The program was divided into two 60-minute training sessions per week, led by an experienced instructor. The first session consisted of a circuit exercise program performed to music in a full group. It included ten stations with various full-body exercises (see Appendix A for the complete list), along with a joint warm-up and cool-down. No external load was used for these exercises. Each station lasted 40 s, followed by a 20-second rest. The participants were instructed to complete as many repetitions as possible at a challenging yet manageable level. For those unable to attend any group session, the program could be performed at home with a written sheet and a video of the exercises provided. The second session was conducted once a week in smaller groups at a gym, and focused on strength and balance training using machines and other equipment. It consisted of eight exercises (leg press, leg curl, latissimus pull-down, seated row, back extension, chest press, hip abduction, and balance exercises) as well as warm-up and cool-down exercises. The participants were instructed to perform strengthening exercises with a weight allowing a maximum of 8–10 repetitions (75–80% of one-repetition maximum, 1RM) before reaching muscle failure, completing two to three sets for each exercise. The participants were provided with a logbook to track their strength training progress, including repetitions, sets, and weights used for each machine. The exercises were individually adjusted and progressed over time.

### Safety and compliance

As a safety precaution, all participants underwent vertebral fracture assessment (VFA), and those with vertebral fractures that had not been assessed or previously treated with bone-specific drugs were excluded from the study. Both exercise interventions were supervised by experienced training instructors to ensure fidelity and adherence to the exercise. The exercises were tailored to each participant’s functional ability and health status. The participants were advised to: (i) start slowly, focusing on learning proper exercise techniques before increasing intensity or weight loads to reduce the risk of injury, (ii) disclose any health conditions that required consideration by the instructors, and (iii) report any injuries or adverse effects related to the exercise. The training instructors recorded participants’ attendance, and participants were provided with a physical activity diary to log their exercise attendance and any physical activity performed outside the scope of the study. Instructions were given to the participants not to alter their physical activity habits during the study period, except to continue any exercise routines they had been following prior to enrollment. This restriction remained in place until the 9-month follow-up visit.

### Data collection

The participants were required to attend two visits to complete all study measurements, which included body composition assessments, questionnaires, and physical tests. Follow-ups were conducted right after the nine-month intervention had ended. Three months into the intervention, there was a short follow-up session conducted by telephone, where the participants were asked about their current health and study status. Data collection began in October 2021 and concluded in July 2023.

### Outcome measures

#### Muscle function and mobility

The assessment of muscle function included several physical tests. Back extensor strength (measured in Newton meters, Nm), was measured isometrically with a testing device (DigiMax ISO-Check, mechaTronic, Germany), where the participants sit strapped into the device and pushes the upper back against the back support pad [15]. Grip strength (measured in kilograms, kg) was measured with JAMAR, both hands, with the participants sitting down, holding the device in approximately 90 degrees of elbow flexion [16]. Isometric trunk extension (measured in seconds) was measured with the Biering-Sorensen’s test, where the participants lay prone on a bench, with their arms crossed over their chest, holding their upper body steady in a horizontal position for as long as possible while the test leader supports the participants’ ankles [17]. Isometric trunk flexion (measured in seconds) was measured with a static sit-up test, with the participant seated on the floor, and their trunk positioned backwards in a 45-degree hip angle, holding that position for as long as possible, while the test leader supported their feet and ankles [18]. The 50 sit-to-stand test (measured in repetitions and seconds) was conducted with participants performing as many sit-to-stand repetitions as quickly as possible, up to a maximum of 50 [18]. The test was performed using a standard chair (45 cm in height) without armrests or hand support. The number of repetitions completed within the first 30 s (30-s sit-to-stand), the total number of repetitions, and the total time in seconds were recorded. Five sit-to-stand (measured in seconds) was performed using the same chair set-up as the 50-sit-to-stand test [19]. Gait speed (measured in meters per second) was measured over a 30-meter distance, where participants walked at their maximum speed over two laps of 15 m [20]. Timed Up and Go (TUG) (measured in seconds) was conducted according to standardized procedure [21].

### Balance

The assessment of balance included both static and dynamic balance tests. One-leg standing test (OLST) (measured in seconds, with a maximum of 60) tests were performed without shoes, with participants completing the test with their eyes open and then closed. The participants were instructed to keep their arms alongside their bodies and position the non-support leg away from the standing leg [22]. The test was stopped if the participants started jumping, put down the other foot, or took support from their hands; or opened their eyes during the test with their eyes closed. Tandem standing test (measured in seconds, with a maximum of 60) was performed with the participants’ putting one foot in front of the other (heel-to-toe, whichever foot in the front they preferred), and having their arms to the side of their body. The test was performed with eyes opened and closed. The test was stopped if the participants moved their feet or took support from their hands. Tandem walking (measured as the number of correct steps, with a maximum of 15) was performed with participants taking heel-to-toe steps forward and backward along taped straight lines (2 cm wide tape) on the floor [23, 24]. When walking forward, the participants were instructed to place their steps directly on top of the line. When walking backward, the participants were told to place their steps in between two lines (15 cm apart). The test was stopped if a participant placed a foot off the line or used their hands for support.

For information about general assessment procedures, please refer to Appendix B.

### Statistical analysis

The statistical analyses in this study were based on outcomes from a randomized controlled trial primarily focused on bone strength, for which the power calculation was conducted [25]. Therefore, no additional power calculation was conducted for these secondary outcomes. As the outcomes were exploratory, no adjustments for multiple comparisons were made. Both intention-to-treat (ITT) and per-protocol (PP) analyses were performed for all outcome measures. A complete-case analysis was conducted, and no imputation was applied due to the low proportion of missing data. Missing data primarily resulted from participant dropouts. For those who attended follow-up assessments, missing data for individual outcomes ranged from 8.8% to 10.3%. There were no systematic differences between participants, suggesting that data were missing at random. One TUG data point at follow-up was removed as an extreme outlier due to a temporary illness that affected the participant’s performance. For the PP analysis, only participants with an attendance rate of at least 70% were included. The normality of continuous variables was assessed by visually inspecting histograms and Q-Q plots to evaluate the approximate normal distribution of the raw data. If the assumptions for parametric tests were violated, non-parametric alternatives were applied.

All outcomes were analyzed by comparing follow-up and baseline values. Data were presented as mean (M) ± standard deviation (SD) for normally distributed continuous variables and median (Md) with interquartile range (IQR) for non-normally distributed continuous variables. Categorical variables were reported as counts (n) and percentages (%). Within-group differences were analyzed using paired samples t-tests, Wilcoxon signed-rank test or McNemar’s chi-square test, while between-group differences were assessed using independent t-tests, Wilcoxon rank sum tests, or Pearson’s chi-square tests, depending on data distribution and variable type. To account for potential confounding and modification effects of age, linear regression models were fitted to the difference in continuous outcome adjusting for age and including an interaction term between age and treatment group. All tests were two-sided, and a p-value ≤ 0.05 was considered significant. All statistical analyses were performed using STATA 18 (StataCorp. 2023. Stata Statistical Software: Release 18. College Station, TX: StataCorp LLC).

### Research ethics

This study received ethical approval from the Swedish Ethical Review Authority (Dnr: 2020–04359). All participants provided written informed consent prior to participation. The study adhered to the principles outlined in the World Medical Association’s Declaration of Helsinki.

## Results

### Enrollment and allocation

A total of 194 women, with a median age of 70 years, were randomly assigned to either the OS group (*n* = 97) or the DME group (*n* = 97). Of these, 168 participants (86.6%) completed the trial, while 26 (13.4%) dropped out, evenly distributed between both groups (13 per group). In total, 178 women (91.7%) took part in the 9-month follow-up. A detailed overview of enrollment and group allocation is presented in Fig. S1 in Supplementary materials. The ITT analysis included all 194 participants, while the PP analysis was conducted with 149. The most common reasons for exclusion before randomization were the inability to attend exercise sessions due to location or scheduling conflicts, recent initiation or discontinuation of bone-specific drug therapy, a T-score above − 1.0, and untreated vertebral fractures.

### Baseline characteristics

The participants had a median age of 70 years, with a mean height of 163.3 cm, weight of 64.5 kg, and BMI of 24.2. There were no significant differences between the groups in any of the baseline characteristics (Table 1). The participants reported an average of 290 min of at least moderate-intensity physical activity per week (with no significant difference between the OS and DME groups). In total, 85% met the WHO recommendation of 150–300 min per week.

**Table 1:** Baseline characteristics of the participants (*n* = 194)

Parameter	OS (*n* = 97)	DME (*n* = 97)
Age (median years, IQR)	70 (67–75)	70 (67–75)
Height (mean cm, SD)	163 ± 6	162 ± 6
Weight (mean kg, SD)	64 ± 9	64 ± 9
Waist (mean cm, SD)	81.9 ± 9.8	81.6 ± 9.2
Hip circumference (mean cm, SD)	98.6 ± 6.6	98.5 ± 7.2
Waist-hip ratio (mean, SD)	0.83 ± 0.1	0.83 ± 0.1
Abdominal height (mean cm, SD)	19.9 ± 2.4	19.9 ± 2.2
BMI (mean kg/m^2^, SD)	24.2 ± 3.4	24.1 ± 3.4
Bone-specific drugs^a^ (n, %)	11 (11%)	12 (12%)
Previous fracture (n, %)	48 (49%)	51 (54%)
FRAX score MOF% (median, IQR)	12 (9.1–19)	13 (8.75-17)
FRAX score hip fracture % (median, IQR)	1.9 (1–4)	1.9 (0.9–3.9)
T-score LS Total (mean, SD)	-1.56 ± 1.25	-1.69 ± 1.15
T-score FN Right (mean, SD)	-0.848 ± 0.846	-0.791 ± 0.786
T-score FN Left (mean, SD)	-0.870 ± 0.762	-0.830 ± 0.708
Meeting WHO’s recommendation of 150–300 min of weekly physical activity (n)^b, c^	87 (90%)	81 (84%)
Weekly physical activity levels overall (minutes)^b^	304.6 ± 125.1	275.5 ± 122.9
Weekly everyday physical exercise levels (minutes)^b^	48.9 ± 45.3	42.1 ± 43.7
Weekly physical activity levels (minutes)^b^	206.9 ± 85.8	191.3 ± 97.9

BMI = body mass index; MOF = major osteoporotic fracture, FRAX = fracture risk assessment tool; LS = lumbar spine; FN = femoral neck; WHO = World Health Organization.

^a^On-going treatment with bisphosphonates (*n* = 23) for at least a year: Alendronate (48%), Zolendronate (35%), Denosumab (17%).

^b^Self-reported physical activity with at least moderate intensity.

^c^Total number of participants meeting the recommendation of 150–300 min of weekly physical activity (moderate intensity) = 168 (87%).

### Effects on muscle function and mobility

**Fig. 1:**
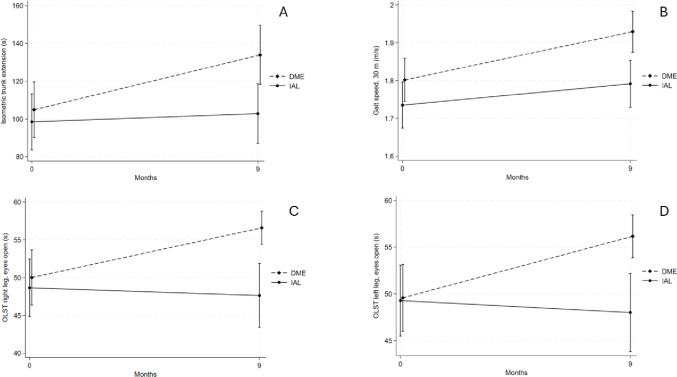
Outcomes showing significant difference between groups: **A**) OLST right leg, eyes open (seconds), **B**) OLST left leg, eyes open (seconds), **C**) Gait speed, 30 m (m/s), **D**) Isometric trunk extension (seconds). OLST = One-leg standing test

Changes in muscle function, mobility, and balance outcomes, along with between-group comparisons from the ITT analysis, are presented in Table 2. At follow-up, the ITT analysis revealed a significantly higher increase in isometric trunk extension for the DME group compared to the OS group (+ 27.6% vs. +4.4%; 95% CI: 118–150 vs. 87–119 s; *p* = 0.007) (Fig. 1). Additionally, both groups showed significant improvements in gait speed, with a significant between-group difference favoring the DME group (DME + 7.1% vs. OS + 3.2%; 95% CI: 1.87–1.98 vs. 1.73–1.85 m per second; *p* = 0.001) (Fig. 1). The OS group demonstrated a significant within-group increase in the number of sit-to-stands in the 30-s sit-to-stand; however, no significant between-group difference was observed. There were within-group improvements in grip strength, back strength, isometric trunk flexion endurance, 5 sit-to-stand and 50 sit-to-stand speed in both intervention groups with no significant differences between the groups. No significant improvements were found in 50 sit-to-stand total numbers performed or TUG in any of the groups.

### Effects on balance

At follow-up, the ITT analysis revealed a significant increase in the OLST (eyes open) for the DME group in both the right (+ 13.1% vs. -2.1%; 95% CI: 54–59 vs. 43–52 s; *p* = 0.000) and left leg (+ 13.3% vs. -2.4%; 95% CI: 54–58 vs. 44–52 s; *p* =< 0.000) compared to the OS group (*p* =< 0.001) (Fig. 1). Additionally, there were significant within-group improvements in tandem walking forward in both groups (OS: +8.1%, *p* = 0.025; DME: +7.4%, *p* = 0.001). A significant between-group difference, likely due to random variation, was present at baseline and persisted at follow-up. No significant differences were observed in OLST (eyes closed), tandem standing (eyes open and closed), or tandem walking backward in either group.

For results from the per protocol analysis and age adjustments, please refer to Appendix C. 

For results related to safety and adherence, please refer to Appendix D.

**Table 2:** Intention-to-treat outcomes at baseline and 9-month follow-up for each group, presented as mean ± SD. Within- and between-group differences were analyzed using paired t-tests and independent t-test for continuous variables, or McNemar’s and Pearson’s chi square test for categorical variables, comparing values to baseline. Significant p-values are highlighted in bold

Outcome	OsteoStrong (*n* = 97)				Dynamic multicomponent exercise (*n* = 97)				
	Baseline	Follow-up	Change	Within-group *p*-value	Baseline	Follow-up	Change	Within-group *p*-value	Between-group *p*-value
Grip strength, right (kg)	23.8 ± 5.0	25.3 ± 4.4	+ 6.4%	**< 0.001**	22.9 ± 4.9	24.1 ± 5.0	+ 4.9%	**< 0.001**	0.084
Grip strength, left (kg)	22.1 ± 4.8	23.6 ± 4.4	+ 7.0%	**< 0.001**	21.4 ± 5.0	22.2 ± 4.9	+ 4.1%	**0.004**	0.057
Back extensor strength (Nm)	117.6 ± 49.3	140.6 ± 45.3	+ 19.5%	**< 0.001**	123.8 ± 49.9	142.2 ± 42.8	+ 14.8%	**< 0.001**	0.808
Isometric trunk extension (sec)	98.4 ± 73.4	102.8 ± 75.3	+ 4.4%	0.485	104.8 ± 73.2	133.8 ± 74.9	+ 27.6%	**< 0.001**	**0.007**
Isometric trunk flexion (sec)	109.8 ± 81.3	135.9 ± 94.3	+ 23.8%	**0.002**	100.5 ± 68.5	134.5 ± 86.5	+ 33.9%	**< 0.001**	0.916
5 sit-to-stand (sec)	7.9 ± 2.3	7.2 ± 2.4	-9.9%	**< 0.001**	7.7 ± 1.8	7.1 ± 1.8	-7.7%	**0.002**	0.848
30-s sit-to-stand (n)	19.8 ± 5.5	21.6 ± 6.2	+ 9.4%	**0.001**	20.5 ± 5.4	21.0 ± 5.8	+ 2.6%	0.071	0.572
50 sit-to-stand speed (n/sec)^a^	0.63 ± 0.20	0.70 ± 0.22	+ 10.1%	**0.001**	0.64 ± 0.20	0.70 ± 0.24	+ 9.2%	**0.001**	0.900
Gait time, 30 m (sec)	18.1 ± 5.4	17.3 ± 3.5	-4.4%	0.087	17.4 ± 6.1	15.8 ± 2.2	-9%	**0.014**	**0.001**
Gait speed, 30 m (m/sec)	1.73 ± 0.30	1.79 ± 0.30	+ 3.2%	**0.001**	1.80 ± 0.29	1.93 ± 0.26	+ 7.1	**< 0.001**	**0.001**
Timed Up and Go (sec)	6.3 ± 1.4	6.2 ± 1.1	-1.2%	0.564	6.0 ± 0.9	5.9 ± 1.0	-1.8%	0.491	0.084
OLST, right, eyes open (sec)b	48.6 ± 18.9	47.6 ± 20.3	-2.1%	0.670	50.0 ± 18.2	56.6 ± 10.6	+ 13.1%	**< 0.001**	**0.005**
OLST, left, eyes open (sec)c	49.3 ± 18.9	48.0 ± 20.0	-2.4%	0.796	49.6 ± 17.9	56.2 ± 11.0	+ 13.3%	**< 0.001**	**0.005**
OLST, right, eyes closed (sec)	10.6 ± 12.8	9.6 ± 11.2	-9.5%	0.184	9.45 ± 10.4	11.3 ± 13.5	+ 18.6%	0.202	0.345
OLST, left, eyes closed (sec)	9.67 ± 9.9	9.1 ± 11.3	-6.1%	0.334	9.1 ± 9.5	10.4 ± 11.0	+ 13.4%	0.245	0.429
Tandem standing, eyes open (sec)d	55.6 ± 13.5	55.8 ± 12.4	+ 2.4%	0.317	57.5 ± 9.5	58.3 ± 7.5	+ 1.4%	0.763	0.111
Tandem standing, eyes closed (sec)	14.6 ± 17.2	15.4 ± 17.4	+ 5.4%	0.976	18.5 ± 19.1	16.1 ± 17.4	-13.8%	0.089	0.771
Tandem walking, forward (steps)e	12.1 ± 4.6	13.1 ± 3.8	+ 8.1%	**0.025**	13.7 ± 3.2	14.7 ± 1.7	+ 7.4%	**0.001**	**< 0.001** ^**b**^
Tandem walking, backward (steps)f	11.3 ± 5.0	11.1 ± 4.8	+ 1.6%	1.000	12.5 ± 4.3	13.0 ± 3.4	+ 3.8%	1.000	0.135

^a^Baseline proportion completing the maximum 50 sit-to-stands: OS = 82% vs. DME = 88% (*p* = 0.719); Overall = 85%.

^b^Baseline proportion completing the maximum 60 s OLST, right, eyes open: OS = 68% vs. DME = 70% (*p* = 0.438); Overall = 69%.

^c^Baseline proportion completing the maximum 60 s OLST, left, eyes open: OS = 69% vs. DME = 68% (*p* = 0.512); Overall = 68%.

^d^Baseline proportion completing the maximum 60 s Tandem standing, eyes open: OS = 83% vs. DME = 91% (*p* = 0.446); Overall = 87%.

^e^Baseline proportion completing the maximum 15 steps Tandem walking, forward: OS = 66% vs. DME = 82% (*p* = 0.087); Overall = 74%.

^f^Baseline proportion completing the maximum 15 steps Tandem walking, backward: OS = 58% vs. DME = 69% (*p* = 0.752); Overall = 63%.

^g^=Also significant between-group difference at baseline.

## Discussion

### Summary of key findings

This study aimed to compare the effects of OsteoStrong^®^ (OS) and dynamic multicomponent exercise (DME) on muscle function, mobility, and balance in older women for a 9-month intervention. Overall, significant between-group differences were observed in back endurance, gait speed, and the balance test OLST (eyes open), all favoring the DME group. Participants in both groups showed significant improvements in grip strength, back extensor strength, isometric trunk flexion, 5 sit-to-stand and 50-sit-to-stand speed at follow-up with no significant between-group differences. Regarding balance, tandem walking forward improved significantly in both groups, while OLST (eyes open) improved significantly only in the DME group.

### Interpretation of the results and comparison to previous research

Given the limited clinical research on isometric exercise, particularly OS, it is interesting that most muscle function tests and gait speed showed significant improvements following the intervention. This is particularly remarkable considering the relatively short duration of time spent performing OS, suggesting that even brief exposures to high-magnitude mechanical loading can yield measurable functional benefits. Similar to bone tissue, muscles exhibit a dose-dependent response to mechanical loading, with higher-magnitude forces eliciting greater adaptations in strength. Previous research has shown that high-load resistance training (≥ 80% of 1RM) is more effective than low-load resistance training (< 60% of 1RM) in improving 1RM strength, although both methods are beneficial for muscle strength and hypertrophy adaptations [26]. High-load resistance training may induce greater neuromuscular adaptations, leading to increased motor unit recruitment and improved force production [27]. In contrast, low-load, high-volume training has been shown to promote muscular endurance and hypertrophy [28]. Additionally, current data suggest that ballistic isometric training also improves neuromuscular activation and force output [7, 8]. Thus, although the OS was brief, the magnitude of loading is likely responsible for the improvements in muscle function. The improved muscle function probably contributed to the increase in gait speed, as there is a correlation between muscle strength and gait speed [29]. Another possible contributing factor to the increase in gait speed is that participants may have increased their walking activity by traveling to the training center throughout the trial, affecting both groups. The observed mean increase in gait speed (OS: +0.06 m/sec; DME: +0.13 m/sec) aligns with the minimal clinically important difference (MCID) of 0.1–0.2 m/sec reported for older adults, underscoring its functional relevance − particularly for the DME group [30]. Interestingly, gait speed showed a significant between-group difference in favor of the DME group, despite no significant between-group differences in 5 sit-to-stand, 30-second sit-to-stand, 50 sit-to-stand speed or TUG, which assesses leg muscle function. We do not know whether other physical activities have been performed by the participants in addition to the short OS sessions during the intervention period that could explain some results for this group.

The significant improvement in isometric trunk extension observed in the DME group compared to the OS group suggests that DME may be more effective in enhancing back extensor muscular endurance. This finding aligns with previous research demonstrating that dynamic resistance exercises are particularly beneficial for muscle endurance adaptations [28]. In contrast, isometric exercise primarily targets maximal strength by eliciting high-intensity contractions for 15–20 s, which may explain the non-significant increase in back endurance in the OS group. Additionally, in contrast to the OS program, the DME program incorporated a variety of exercises targeting the back muscles, including both machine-based and bodyweight movements, providing a more comprehensive stimulus for muscular endurance.

The significant between-group difference in the one-leg standing (OLS) test, favoring the DME group, suggests that dynamic multicomponent exercise is more effective in improving static balance compared to OS. This finding is consistent with previous research indicating that balance training, particularly when combined with dynamic and weight-bearing exercises, enhances neuromuscular control and postural stability [6]. The DME program incorporated various exercises that challenged balance, including weight shifts, step-ups, coordinated movements, and lower-limb strengthening, which likely contributed to the observed improvements. In contrast, although the OS group performed isometric contractions with high force, these exercises primarily targeted muscle strength rather than the complex sensorimotor adaptations required for balance control. Additionally, while the OS group practiced one-leg standing exercises on a vibration platform only once a week, the DME group performed other balance exercise tasks twice a week. Balance is a critical factor in fall prevention, particularly in older adults with osteoporosis, as impaired balance increases the risk of falls and fractures [31–33]. These results underscore the importance of incorporating dynamic balance exercises into osteoporosis management programs. Currently, no MCID has been established for the OLST [34].

The relatively high baseline physical activity levels among participants, with 87% being physically active compared to the national average of 54% for Swedish women aged 65–84 years [35], may have influenced the study results, potentially attenuating the observed effects of both interventions. Since regular physical activity is associated with better physical functions, the participants may have already possessed a higher level of physical fitness than the general population of older women with osteopenia or osteoporosis. This could have resulted in a “ceiling effect”, where the potential for further improvement was limited, particularly in outcome measures such as gait speed, sit-to-stand performance, and balance tests. This factor might explain why some outcome measures showed only modest improvements or lacked significant between-group differences. For comparison with normative data, the mean gait speed at baseline was 1.77 m/s in our study (for both groups together), compared to 1.54 m/s shown in another study of Swedish older women [36]. The mean TUG time at baseline was 6.2 s, which is similar to a study from Brazil reporting 5.6–8.4 s [37], whereas a study on Iranian women aged 60–79 years reported approximately 10.7 s [38]. For the static sit-up test (isometric trunk flexion 45^◦^), the mean baseline time was 105.1 s, compared to 68 s in Swedish women aged 65–80 years [18]. Similar baseline comparisons between our results versus the latter study were for 50 sit-to-stand speed 0.64/s vs. 0.53/s; 30s sit-to-stands 20 vs. 17; five sit-to-stand 7.8s vs. 10.3s; trunk extension endurance 102s vs. 80s; balance test OLST (eyes open, both legs summarized) 49s vs. 39s; OLST (eyes closed) 9.7s vs. 8.0s [18]. Lastly, the mean baseline grip strength (right hand) in our study was 23.4 kg, comparable to 23.5 kg in a separate cohort of Swedish women and higher than the approximately 19.6 kg observed in Polish women aged 65–79 years [39, 40].

Both groups demonstrated a high attendance rate, with the OS group showing a higher rate. As anticipated, the PP analysis revealed greater improvements in muscle function tests within the DME group compared to the ITT analysis, particularly in the 30-s sit-to stand, 50 sit-to-stand speed, and back strength. Since most participants in the OS group were already highly adherent in the ITT analysis, the PP analysis did not reveal additional improvements in outcomes beyond those initially observed. This suggests that higher adherence to dynamic multicomponent exercise may enhance strength and functional outcomes. However, differences in exercise fidelity and supervision between the groups may have influenced the results. The OS group performed their exercises under close supervision with real-time feedback from machines displaying force output, ensuring that participants reached the target intensity in each session. In contrast, the DME sessions were conducted in larger groups, which may have limited the ability to provide personalized supervision and adjustments, potentially affecting exercise execution and progression. These factors emphasize the importance of exercise adherence and monitoring in maximizing its benefits.

To our knowledge, only one other clinical trial has evaluated the effects of a machine-based OS system similar to ours on physical functions [10]. That 8-month intervention study compared high-intensity resistance and impact training (HiRIT), machine-based isometric axial compression (IAC) (BioDensity™), and a control group, which included 93 male participants (mean age of 67 years). The study found that the HiRIT group achieved the best outcomes in BMD and physical function compared to the IAC and control groups [10]. The HiRIT group had a significant improvement in 5 sit-to-stand (decreased time) compared to the IAC group (-10.7% vs. -4.5%; *p* = 0.01). Additionally, the HiRIT had a significant improvement in peak impulse (N·s) and peak impulse relative to body weight (N·s/kg) compared to the IAC group. The HiRIT group also had a significant increase in leg extensor strength compared to the control group, whereas the IAC did not; however, there were no significant differences between the HiRIT and IAC. The IAC group showed improved muscle function (five-times sit-to-stand) compared to the control group [10]. In our study, both groups showed significant improvements in 5 sit-to stand; however, no significant difference was observed between the groups. Although our study intervention lasted one month longer, the study by Harding and colleagues had a more intensive exercise regimen in the HiRIT group compared to our DME program, which may explain why they observed a group difference while we did not. Additionally, their program included a jumping chin-up exercise, likely enhancing participants’ muscle power – a key factor in performing the 5 sit-to-stand test [41].

DME resulted in superior improvements in balance, back endurance, and gait speed. The DME intervention was of longer duration than the OS intervention. It is possible that the improvements in the OS group would have been greater with a higher exercise frequency and longer intervention duration. However, the once‑weekly loading program represents the standardized protocol offered by OS to its members. Nevertheless, OS also provided significant gains in some tests regarding muscle function and mobility. Given its lower time commitment and high adherence rate, OS may be a practical alternative for individuals with mobility limitations or those who struggle with more complex movement patterns required in DME. Additionally, the OS training concept could appeal to individuals hesitant to join traditional gyms. Future research should explore whether a combination of isometric and dynamic training could optimize strength, mobility, and balance outcomes. Another factor to consider in this context is whether the individuals in the respective groups (OS and DME) performed other physical activity in addition to the organized exercise and if so, to what extent and with what intensity. It could be that the individuals in the OS group, in addition to the weekly leader-led training of 20 min, have performed other physical activities that may have affected the fitness test results. One way to study this in more detail is to use the objective measurement method of accelerometry in future studies.

### Strengths and limitations

There are several strengths of this study. It boasts a relatively large sample size, ensuring robust statistical power. The comprehensive evaluation of physical functions provides a detailed assessment of the participants’ capabilities. Furthermore, the high attendance rate and relatively low dropout rate enhance the reliability of the findings. Another notable strength is the consistency in assessment, with all but one participant evaluated by the same physical therapist at both baseline and follow-up. Finally, the study’s design as a randomized controlled trial is a significant strength, offering a structured approach to producing reliable results. The study also has some limitations. One limitation is the absence of a formal power calculation, as the analyzed outcomes were secondary endpoints from a previously conducted randomized controlled trial. However, prior studies with smaller sample sizes have reported similar findings, suggesting that the study was likely sufficiently powered to detect meaningful differences. Nonetheless, as with any secondary analysis, these results should be interpreted with caution, and future studies specifically designed to assess these outcomes will help strengthen the evidence base. Another limitation is that the physical therapists who conducted the assessments were not blinded at follow-up. However, they were blinded at baseline, were not involved in the exercise intervention, and conducted all tests in a standardized manner. A third limitation is the absence of repeated one-repetition maximum (1RM) tests to monitor exercise fidelity in the DME group. Regular 1RM assessments could have provided a more precise measure of participants’ strength progression, ensuring that exercise intensities were consistently aligned with their capabilities throughout the intervention. However, due to practical challenges, including time and resource constraints, this was not possible for us to perform.

## Conclusion

This randomized controlled trial demonstrated that both OsteoStrong^®^ (OS) and dynamic multicomponent exercise (DME) improve muscle function, mobility, and balance in older women with osteopenia or osteoporosis. However, DME resulted in significantly greater improvements in gait speed, back endurance, and static balance compared to OS. The high adherence rates in both groups, along with the low number of adverse events, suggest that both exercise modalities were well tolerated among the older women in this study. These findings indicate that OS may serve as an alternative or adjunct for individuals who find dynamic exercise too physically demanding, yet DME remains the more effective option for improving overall functional capacity. However, it is important to also consider the time requirements of each intervention when evaluating their suitability. These findings should be interpreted with caution, as they are based on secondary outcomes. Given the importance of muscle strength, mobility, and balance in osteoporosis management, healthcare professionals should tailor exercise recommendations based on individual needs, physical capacity, and preferences. Further studies are needed to confirm these findings and refine exercise recommendations.

## Supplementary Information

Below is the link to the electronic supplementary material.

Supplementary Material 1

Supplementary Material 2

Supplementary Material 3

Supplementary Material 4

Supplementary Material 5

Supplementary Material 6

## Data Availability

Data from this study are available upon reasonable request to the corresponding author after an assessment of confidentiality due to ethical and legal restrictions according to national legislation.
