# Supplementary figures and images for: Effects of OsteoStrong vs. dynamic multicomponent exercise on physical function in older women in the BONEMORE randomized controlled trial
Source: Aging Clin Exp Res. 2026 Jul 5;38(1):168. doi: 10.1007/s40520-026-03421-4 (PMC13424000; doi:10.1007/s40520-026-03421-4)

## CONSORT 2010 Flow Diagram

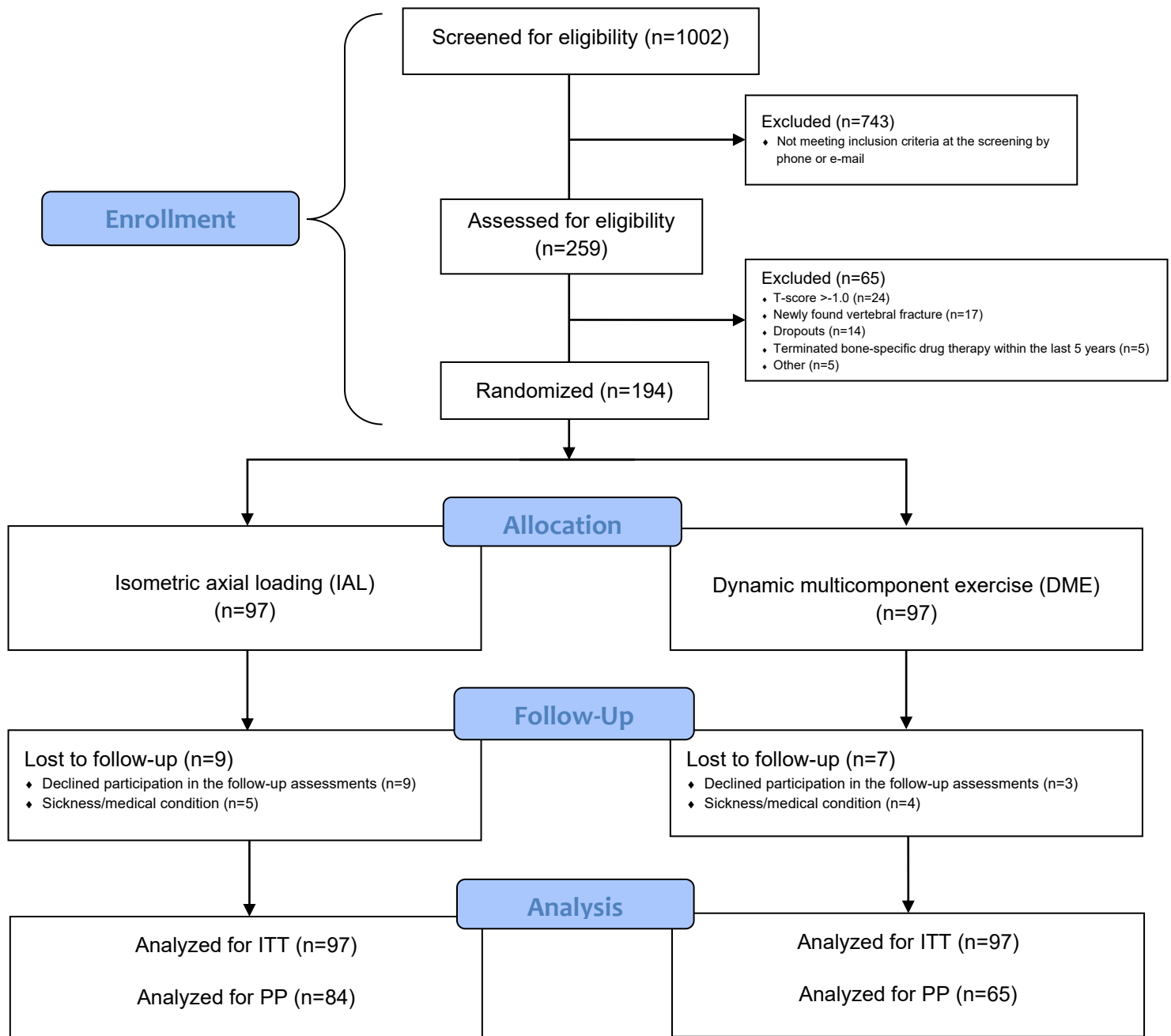

Fig 1. CONSORT study flow diagram. ITT = Intention-to-treat; PP = Per-protocol.

Supplement: Supplementary file 1 — Supplementary Material 1 [file 40520_2026_3421_MOESM1_ESM.pdf]
