# Supplementary material for: Effects of OsteoStrong vs. dynamic multicomponent exercise on physical function in older women in the BONEMORE randomized controlled trial
Source: Aging Clin Exp Res. 2026 Jul 5;38(1):168. doi: 10.1007/s40520-026-03421-4 (PMC13424000; doi:10.1007/s40520-026-03421-4)
Supplement: Supplementary file 2 — Supplementary Material 2 [file 40520_2026_3421_MOESM2_ESM.pdf]

Appendix A. Dynamic Multicomponent Exercise list

All exercises were individually adapted to the participants' physical status and capabilities.

Session 1: Circuit exercise program (60 minutes)

Exercises 2-11 were performed for 40 seconds in three sets

1. Warm-up exercises (light movements and stretches)
2. Squats
3. Side-lying hip raise
4. Supine hip raise
5. Skater jumps
6. Push-ups
7. Light jumps while walking
8. "Dead bug" (core exercise)
9. Prone back extensions
10. Step up
11. Balance exercises (e.g. one-leg standing, standing on a balance board)
12. Cool-down exercises (light movements and stretches)

Session 2: Strength training at the gym (60 minutes)

Exercises 2-9 were performed 8-10 repetitions in three sets

1. Warm-up exercises (e.g. walking on a treadmill, indoor bicycle)
2. Leg press
3. Leg curl
4. Latissimus pull-down
5. Seated row
6. Back extension (belly-back)
7. Chest press
8. Hip abduction (standing or side-lying)
9. Balance exercises
10. Cool-down exercises (light movements and stretches)
