# Supplementary material for: Effects of OsteoStrong vs. dynamic multicomponent exercise on physical function in older women in the BONEMORE randomized controlled trial
Source: Aging Clin Exp Res. 2026 Jul 5;38(1):168. doi: 10.1007/s40520-026-03421-4 (PMC13424000; doi:10.1007/s40520-026-03421-4)
Supplement: Supplementary file 3 — Supplementary Material 3 [file 40520_2026_3421_MOESM3_ESM.pdf]

Appendix B. General Assessment Procedures and Anthropometric Measurements

General assessment procedure

All tests were demonstrated by the test leader prior to the start. The participants were given the opportunity to practice the test beforehand and were instructed to perform the tests to the best of their ability while prioritizing safety. Each test was performed at least twice, except for the most physically demanding tests (isometric trunk extension, isometric trunk flexion, and 50 sit-to-stand), or when participants achieved the maximum score on the balance tests. The best score from each test was included in the statistical analysis. All tests were conducted by two test leaders (PL and CKA), who generally assessed the same participants at both baseline and follow-up.

Assessment of anthropometry

Height was measured using a wall-mounted stadiometer, and body weight was recorded using a calibrated digital scale. Body mass index (BMI) was calculated as weight (kg) divided by height squared (m^2). Waist and hip circumferences were measured with a flexible measuring tape to evaluate body fat distribution. All measurements were conducted following standardized procedures to ensure accuracy and reliability.
