# Supplementary material for: Effects of OsteoStrong vs. dynamic multicomponent exercise on physical function in older women in the BONEMORE randomized controlled trial
Source: Aging Clin Exp Res. 2026 Jul 5;38(1):168. doi: 10.1007/s40520-026-03421-4 (PMC13424000; doi:10.1007/s40520-026-03421-4)
Supplement: Supplementary file 4 — Supplementary Material 4 [file 40520_2026_3421_MOESM4_ESM.docx]

**Appendix C. Per-Protocol Results and Age Adjustments**

**Effects on muscle function and mobility**

The PP analysis revealed some differences compared to the results from the ITT analysis (see Table 3). The 30-s sit-to-stand was significant in the DME group, increasing from +2.6% to +6.3% (p=0.002). In the OS group, 50 sit-to-stand speed was no longer significant, changing from +10.1% to -5.5% (p=0.481). Additionally, the DME group showed greater improvements in back strength (+20.1% vs. +14.8%), 50 sit-to-stand speed (+24.5% vs. +5.9%) compared to the ITT analysis. Other outcomes did not differ considerably from the ITT analysis. The linear regression showed that older age at baseline was significantly associated with lower muscle strength and mobility performance in several outcomes (e.g., grip strength, back extensor strength, and gait speed; p<0.05). However, adjusting for age did not alter the significance of the main findings. No significant interaction between treatment and age was found.

**Effects on balance**

The PP analysis revealed a slightly higher percentage 10.1% (p=0.001) in tandem walking forward for the OS group. The DME group showed a lower percentage, 8.5% (p=0.013), in OLST (eyes open) on the right leg. However, a significant between-group difference still favored the DME group. Other outcomes did not differ significantly from the ITT analysis. Linear regression revealed that older baseline age correlated with poorer balance across all tests. However, age adjustment did not impact the main findings' significance. No statistically significant interaction between treatment and age was found except for tandem standing, eyes open and tandem walking (forward). For tandem standing (eyes open), performance declined by 0.95 seconds per year of age, while for tandem walking forward, performance decreased by 0.26 correct steps per year of age

**Table S1.** **Per-protocol** (including participants with at least 70% attendance rate) outcomes at baseline and follow-up for each group, presented as mean ± SD. Within- and between-group differences were analyzed using paired t-tests and independent t-test for continuous variables, or McNemar’s and Pearson’s chi square test for categorical variables, comparing values to baseline. Significant p-values are highlighted in bold.

|  | Isometric axial loading  (n=84) | | | | Dynamic multicomponent exercise  (n=65) | | | |  |
| --- | --- | --- | --- | --- | --- | --- | --- | --- | --- |
| Outcome | Baseline | Follow-up | Change | Within-group  p-value | Baseline | Follow-up | Change | Within-group  p-value | Between-group  p-value |
| Grip strength, right (kg) | 24.0 ± 5.1 | 25.5 ± 4.4 | +6.2% | **<0.001** | 23.2 ± 4.9 | 24.2 ± 5.0 | +4.5% | **<0.001** | 0.096 |
| Grip strength, left (kg) | 22.2 ± 4.9 | 23.7 ± 4.4 | +6.8% | **<0.001** | 21.6 ± 4.4 | 22.4 ± 4.4 | +3.8% | **0.001** | 0.074 |
| Back extensor strength (Nm) | 119.7 ± 48.5 | 140.6 ± 46.2 | +17.5% | **<0.001** | 121.6 ± 49.4 | 142.5 ± 43.9 | +17.2% | **<0.001** | 0.799 |
| Isometric trunk extension (sec) | 100.1 ± 74.0 | 105.9 ± 74.7 | +5.8% | 0.229 | 113.5 ± 77.5 | 141.7 ± 72.7 | +24.9% | **<0.001** | **0.004** |
| Isometric trunk flexion (sec) | 112.9 ± 84.4 | 138.9 ± 94.9 | +23.1% | **0.003** | 105.7 ± 69.1 | 140.5 ± 88.4 | +33.0% | **<0.001** | 0.918 |
| 5 sit-to-stand (sec) | 7.7 ± 2.2 | 7.0 ± 2.3 | -9.1% | **<0.001** | 7.6 ± 1.7 | 6.9 ± 1.7 | -9.8% | **<0.001** | 0.678 |
| 30-sec sit-to-stand (n) | 20.2 ± 5.4 | 22.0 ± 6.1 | +8.7% | **<0.001** | 20.5 ± 4.8 | 21.8 ± 5.9 | +6.3% | **0.002^b^** | 0.835 |
| 50 sit-to-stand (n) | 46.9 ± 8.9 | 47.0 ± 8.6 | +0.3% | 0.817 | 48.3 ± 6.2 | 48.5 ± 6.1 | +0.4% | 1.000 | 0.247 |
| 50 sit-to-stand speed (n/sec) | 0.61 ± 0.21 | 0.57 ± 0.19 | -5.5% | 0.481**^a^** | 0.53 ± 0.08 | 0.66 ± 0.13 | +24.5% | **0.003** | 0.144 |
| Gait, 30 m (sec) | 18.2 ± 5,7 | 17.2 ± 3.5 | -5.5% | 0.073 | 16.7 ± 2.1 | 15.6 ± 1.8 | -6.4% | **<0.001** | **0.002** |
| Gait speed, 30 m (m/sec) | 1.74 ± 0.32 | 1.80 ± 0.29 | +3.7% | **0.004** | 1.83 ± 0.23 | 1.95 ± 0.24 | +6.6% | **<0.001** | **0.002** |
| Timed Up and Go (sec) | 6.2 ± 1.5 | 6.1 ± 1.1 | -1.2% | 0.825 | 6.0 ± 1.0 | 5.8 ± 0.9 | -2% | 0.281 | 0.078 |
| OLST, right, eyes open (sec) | 49.6 ± 17.9 | 49.5 ± 18.6 | -0.1% | 0.819 | 51.9 ± 16.1 | 56.3 ± 10.5 | +8.5% | **0.013** | **0.040** |
| OLST, left, eyes open (sec) | 50.6 ± 18.3 | 49.7 ± 18.8 | -1.6% | 0.782 | 49.0 ± 17.8 | 56.1 ± 11.5 | +14.4% | **0.001** | **0.022** |
| OLST, right, eyes closed (sec) | 11.4 ± 13.5 | 9.9 ± 11.4 | -13% | 0.185 | 9.5 ± 10.3 | 10.7 ± 13.7 | +13.1% | 0.396 | 0.704 |
| OLST, left, eyes closed (sec) | 10.1 ± 10.4 | 9.4 ± 11.5 | -6.9% | 0.379 | 8.3 ± 8.2 | 10.3 ± 10.8 | +23.5% | 0.059 | 0.639 |
| Tandem standing, eyes open (sec) | 55.0 ± 13.2 | 56.3 ± 11.3 | +2.4% | 0.782 | 58.0 ± 8.1 | 58.5 ± 7.1 | +0.1% | 1.000 | 0.291 |
| Tandem standing, eyes closed (sec) | 15.6 ± 18.2 | 15.9 ± 17.7 | +1.7% | 0.994 | 17.9 ±18.8 | 14.9 ± 16.6 | -16.9% | 0.131 | 0.730 |
| Tandem walking, forward (steps) | 12.1 ± 4.6 | 13.3 ± 3.7 | +10.1% | **0.012** | 13.9 ± 3.0 | 14.8 ± 1.3 | +6.7% | **0.003** | **<0.001** |
| Tandem walking, backward (steps) | 11.3 ± 5.0 | 11.4 ± 4.7 | +0.2% | 0.655 | 12.7 ± 4.3 | 13.0 ± 3.4 | +2.1% | 0.346 | 0.263 |

^a^=Was significant in the ITT analysis

^b^=Was not significant in the ITT analysis
