# Supplementary material for: Effects of OsteoStrong vs. dynamic multicomponent exercise on physical function in older women in the BONEMORE randomized controlled trial
Source: Aging Clin Exp Res. 2026 Jul 5;38(1):168. doi: 10.1007/s40520-026-03421-4 (PMC13424000; doi:10.1007/s40520-026-03421-4)
Supplement: Supplementary file 6 — Supplementary Material 6 [file 40520_2026_3421_MOESM6_ESM.docx]

**Appendix D. Results related to safety and adherence**

Out of the 194 women who were randomized, 168 successfully completed the 9-month exercise intervention, while 26 participants (OS: 13, DME: 13) dropped out. The primary reasons for dropout included injuries or illness (OS: 5, DME: 11), dissatisfaction with the training (OS: 3, DME: 0), and other unspecified reasons (OS: 5, DME: 2). The average attendance rate was 94% for the OS group and 81% for the DME group, with 3.5% of the latter's sessions being performed at home; these rates exclude dropouts. Among the OS participants, the proportion of women who met or exceeded the trigger level on each training machine was 68% for the upper growth trigger, 87% for the lower growth trigger, 92% for the core growth trigger, and 92% for postural growth trigger. A total of 32 adverse events (AEs) were reported, 18 of which occurred outside the study. Fourteen AEs (OS: 9, DME: 5; Fisher’s exact p=0.406) were directly related to the exercise intervention. The most frequently reported AE was a gradual onset of musculoskeletal pain (n=9), followed by sudden onset pain (n=3), one case of dizziness, and one fall. In five out of the 12 musculoskeletal pain cases, participants had a prior history of pain in the affected area. One serious AE was recorded: a participant in the DME group fell off a balance board, sustaining a fracture of the os triquetrum. At baseline, 21 women had vertebral fractures (OS: 10, DME: 11; χ² p=0.817). After nine months, four new vertebral fractures were identified (OS: 3, DME: 1). Two of these (one in each group) were confirmed to have resulted from accidents unrelated to the study, while the cause of the remaining two fractures (both in the OS group) could not be determined. At follow-up, a total of 31 self-reported falls occurred within the past six months (OS: 17, DME: 14), with no significant difference between the groups (p=0.835).
